# Automated Sequential Derivatization for Gas Chromatography-[Orbitrap] Mass Spectrometry-based Metabolite Profiling of Human Blood-based Samples

**DOI:** 10.21769/BioProtoc.5196

**Published:** 2025-03-05

**Authors:** Akrem Jbebli, Kateřina Coufalíková, Moira Zanaboni, Manuela Bergna, Renzo Picenoni, Jana Klánová, Elliott J. Price

**Affiliations:** 1 RECETOX, Faculty of Science, Masaryk University, Kotlarska 2, Brno, Czech Republic; 2 EIRENE-CZ, Brno, Czech Republic; 3 Thermo Fisher Scientific, Milan, Italy; 4 CTC Analytics AG, Zwingen, Switzerland

**Keywords:** Automation, Derivatization, Thermo Scientific^TM^ TriPlus^TM^ RSH, Metabolite profiling, Gas chromatography–mass spectrometry (GC–MS)

## Abstract

Many small molecules require derivatization to increase their volatility and to be amenable to gas chromatographic (GC) separation. Derivatization is usually time-consuming, and typical batch-wise procedures increase sample variability. Sequential automation of derivatization via robotic liquid handling enables the overlapping of sample preparation and analysis, maximizing time efficiency and minimizing variability. Herein, a protocol for the fully automated, two-stage derivatization of human blood–based samples in line with GC–[Orbitrap] mass spectrometry (MS)-based metabolomics is described. The protocol delivers a sample-to-sample runtime of 31 min, being suitable for better throughput routine metabolomic analysis.

Key features

• Direct and rapid methoximation on vial followed by silylation of metabolites in various blood matrices.

• Measure ~40 samples per 24 h, identifying > 70 metabolites.

• Quantitative reproducibility of routinely measured metabolites with coefficients of variation (CVs) < 30%.

• Requires a Thermo Scientific^TM^ TriPlus^TM^ RSH (or comparable) autosampler equipped with incubator/agitator, cooled drawer, and automatic tool change (ATC) station equipped with liquid handling tools.

Graphical overview

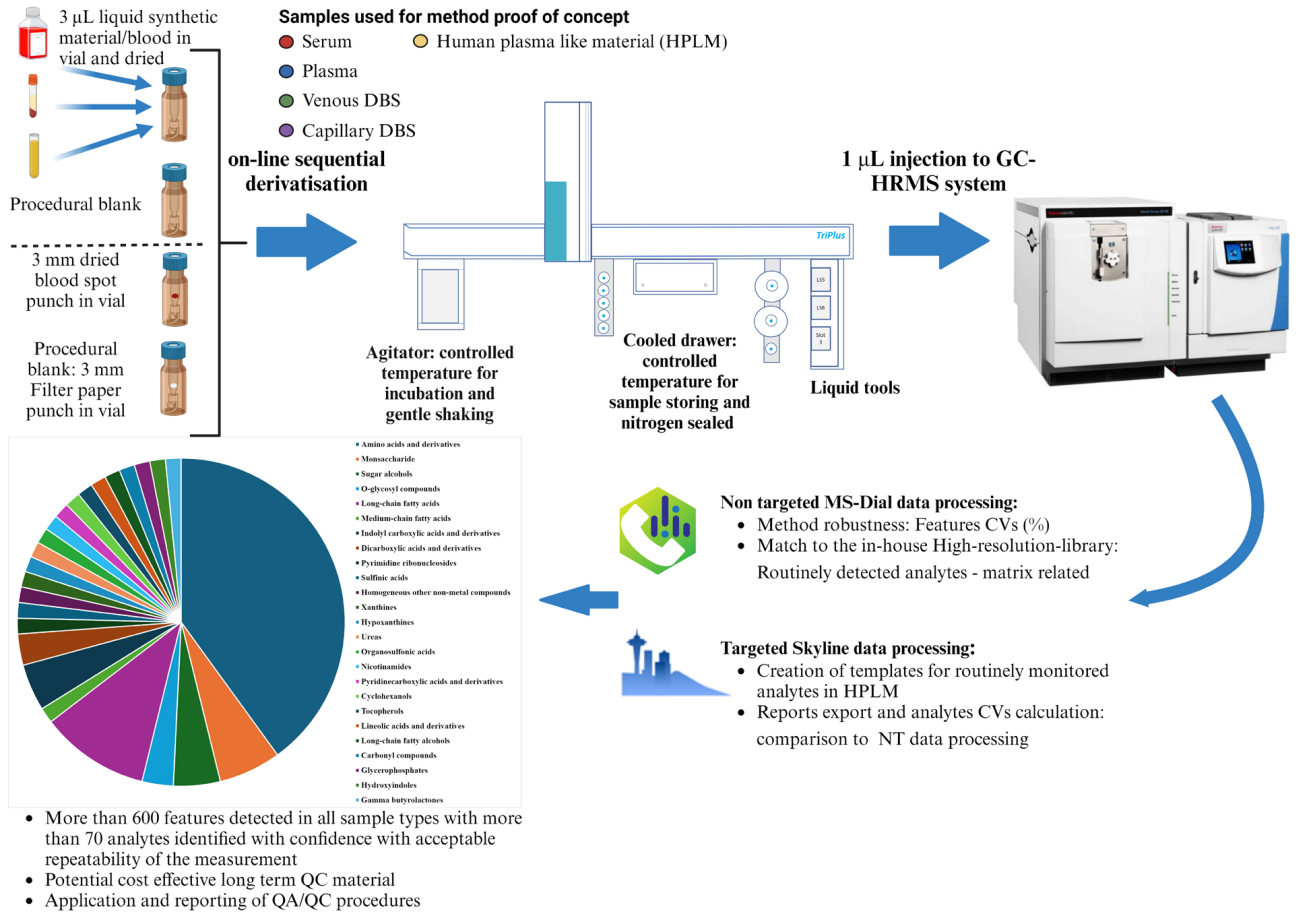

Workflow for profiling metabolites in human blood using automated derivatization

## Background

Gas chromatography–mass spectrometry (GS–MS)-based blood metabolite profiling is an established biochemical technique to investigate human disease and treatment response [1,2], being scalable to large epidemiological studies [3]. Most analysis is conducted on plasma and serum routinely collected in clinical settings, but the use of dried blood spots is prevalent for newborn screening [4] and forensic toxicology, increasingly being collected outside clinical settings for population studies [5].

Once samples are in the lab, sample preparation often constitutes more than 60% of total analytical time [6,7], representing the major bottleneck of throughput. Manual handling during sample preparation contributes to higher bias, with increasing sample-to-sample variation that affects the results and biological interpretation [8].

Derivatization is often employed to make polar non-volatile compounds amenable to GC–MS analysis [9,10]. In human studies, the most used derivatization method for the detection of metabolites is methoximation followed by silylation. Methoximation stabilizes the carbonyl group of, e.g., reducing sugars, whilst silylation acts upon a diversity of functional groups (alcohols, carboxylic acids, amines, thiols, and phosphates), enabling the detection of a broad range of metabolites. Additionally, available electron ionization (EI) MS libraries comprise a high proportion of spectra for trimethylsilyl (TMS) derivatives [11,12] to aid annotation.

However, classical batch-wise derivatization approaches often lead to high sample variability, particularly because of the differing length of time following derivatization until injection. Notably, many derivatization reactions do not have a defined endpoint, and derivatives are often unstable. Furthermore, many analytes form multiple derivatives and isomers [13,14], complicating comparative quantification due to inconsistent ratios of different derivative forms.

To overcome the variability of batch-wise derivatization, procedures for automated, sequential derivatization for human plasma GC–MS metabolomics have been described [15,16]. However, both reported applications had relatively long GC runtimes (>40 min) and sample-to-sample runtimes of ~1 h or more. Additionally, their application was not demonstrated for other blood matrices.

Herein, we describe an automated method for the methoximation-silylation derivatization of metabolites for the sequential, online GC-[Orbitrap] MS-based metabolite profiling of various dried blood matrices, i.e., dried blood spots of capillary and venous blood, dried serum and plasma, and synthetic blood. The sequential sample preparation standardizes the time from derivatization to sample injection, increasing robustness over batch-wise procedures [17], and provides reproducible measurements of ~70 confirmed metabolites per sample with a throughput of ~45 injections per 24 h.

## Materials and reagents

**Biological materials**

1. Dried capillary blood spots (DBS capillary): Capillary blood was collected using a lancet from the finger and spotted onto Whatman 903 protein saver cards [18]. DBS-capillary spots were dried for 12 h at 20 °C in a fume hood. Dried spots were covered with aluminum foil, placed in a zip-lock bag with silica bags to absorb moisture, and stored at -80 °C

2. Dried venous blood spots (DBS venous): Venous blood was collected via S-Monovette^®^ neutral Z (Sarstedt, catalog number: 04.1926.001) [18]. DBS-venous spots were made by dispensing 100 μL onto collection cards following the same procedure as for DBS capillary

3. Gibco human plasma-like material (HPLM) (Thermo Scientific, catalog number: A4899101)

4. Pooled human serum: Mixture of serum derived from patients of University Hospital Brno

5. Pooled human plasma: Mixture of plasma derived from patients of University Hospital Brno

**Reagents**

1. Chloroform (Sigma-Aldrich, catalog number: 65049)

2. Isooctane (Sigma-Aldrich, catalog number: 32291-M)

3. Methanol (Biosolve, catalog number: 136841)

4. Hexane (J.T. Baker, catalog number: 5274.2500)

5. Methoxylamine hydrochloride (Thermo Scientific, catalog number: A19188)

6. N-Methyl-N-trimethylsilyltrifluoroacetamide (MSTFA), ≥98.5% (Sigma-Aldrich, catalog number: 69479-25ML)

7. Pyridine anhydrous, 99.8% (Sigma-Aldrich, catalog number: 270970-100ML)

8. Acetone, ≥99.5% (J.T. Baker, catalog number: 15548454)

9. C_7_-C_40_ saturated alkanes standard, certified reference material (CRM) (MilliporeSigma^TM^ Supelco^TM^, catalog number: 11-101-7207)

10. Fully resolved native Mono-Deca PCB mixture (unlabeled) (Cambridge Isotope Laboratories, Inc., catalog number: EC-5434)

11. D_7_-cholesterol, >99% (Sigma-Aldrich, catalog number: 700041P-10MG)

12. D_5_-glycine, 99% (Sigma-Aldrich, catalog number: 175838-5G)

13. D_27_-myristic acid, 99% (Sigma-Aldrich, catalog number: 366889-1G)

**Solutions**

1. C_7_-C_40_ alkanes solution (1 μg/mL) (see Recipes)

2. Mono-Deca PCBs solution (100–200 ng/mL) (see Recipes)

3. D_7_-Cholesterol stock solution (100 μg/mL) (see Recipes)

4. D_5_-Glycine stock solution (100 μg/mL) (see Recipes)

5. D_27_-Myristic acid stock solution (100 μg/mL) (see Recipes)

6. Internal standard mix (IS mix, 1.6 μg) (see Recipes)

7. Methoximation solution (MeOx, 15 mg/mL, 1 μg/mL ISs) (see Recipes)

8. MSTFA working solution (see Recipes)

**Recipes**

**1. C_7_-C_40_ alkanes solution (1 μg/mL)**

The CRM C_7_-C_40_ saturated alkanes standard contains each alkane at 1,000 μg/mL in hexane. The mixture is diluted in isooctane to achieve a final concentration of 1 μg/mL per each alkane component in a 1 mL final solvent volume. Stored at -20 °C, the solution should be stable for up to a year. However, due to multiple freeze-thaw cycles, we recommend preparing fresh solution every 6 months.

**2. Mono-Deca PCBs solution (100–200 ng/mL)**

The fully resolved native Mono-Deca PCB mixture (unlabeled) is composed of components at 1,000–2,000 ng/mL in isooctane. The mixture is diluted 10-fold to give a solution of components at 100–200 ng/mL in 1 mL of isooctane. Stored at -20 °C, the solution should be stable for up to a year. However, due to multiple freeze-thaw cycles, we recommend preparing fresh solution every 6 months.

**3. D_7_-Cholesterol stock solution (100 μg/mL)**

D_7_-Cholesterol stock solution (100 μg/mL) was prepared by dissolving 10 mg of D_7_-Cholesterol in 1 mL of chloroform, aliquoting 100 μL into 2 mL amber vials (i.e., 100 μg aliquots), and drying for 20 min via centrifugal evaporator operating the low boiling point (bp) program for solvents bp 60–100 °C, without light. Prior to use, 100 μg/mL stock solution is made by reconstitution in 1 mL of chloroform.

**4. D_5_-Glycine stock solution (100 μg/mL)**

D_5_-Glycine stock solution (100 μg/mL) is prepared by dissolving 10 mg of D_5_-Glycine in 1 mL of methanol, aliquoting 100 μL into 2 mL amber vials (i.e., 100 μg aliquots), and drying for 20 min via centrifugal evaporator operating the low boiling point (bp) program for solvents bp 60–100 °C, without light. Prior to use, 100 μg/mL stock solution is made by reconstitution in 1 mL of methanol.

**5. D_27_-Myristic acid stock solution (100 μg/mL)**

D_27_-Myristic acid stock solution (100 μg/mL) is prepared by dissolving 10 mg of D_27_-Myristic acid in 1 mL of chloroform, aliquoting 100 μL into 2 mL amber vials (i.e., 100 μg aliquots), and drying for 20 min via centrifugal evaporator operating the low boiling point (bp) program for solvents bp 60–100 °C, without light. Prior to use, 100 μg/mL stock solution is made by reconstitution in 1 mL of chloroform.

**6. Internal standard mix (IS mix, 1.6 μg)**

Internal standard mix is prepared by dispensing 16 μL of each prepared 100 μg/mL stock standard solution (D_7_-Cholesterol, D_5_-Glycine, and D_27_-Myritic acid) in 2 mL amber vials. The mix is evaporated to dryness via centrifugal evaporator using the same conditions as previously mentioned (~20 min, no light, and low bp program). Vials are capped with the bonded PTFE/silicone septum screw cap and stored at -80 °C.

*Note: Each individual labeled standard stock solution and the IS mix listed above are stored dried at -80 °C; in our experience, they remain stable for >6 months. We recommend periodic monitoring by GC analysis to check stability.*

**7. MeOx solution (15 μg/mL, 1 μg/mL ISs)**

A MeOx stock (15 μg/mL) is freshly prepared every two days via weighing 150 mg of methoxylamine hydrochloride into a 10 mL amber flat-bottom glass vial and dissolving in 10 mL of pyridine. For use, 1.6 mL of MeOx stock is dispensed into the IS mix vials (2–3), capped with pre-slit caps, and vortexed (10 s, 2,000 rpm) to give MeOx solution (15 μg/mL, 1 μg/mL ISs). MeOx solution (15 μg/mL, 1 μg/mL ISs) is placed in the cooled drawer of the autosampler with a controlled temperature at 5 °C and sealed under nitrogen.

**8. MSTFA working solution**

1.6 mL of MSTFA is dispensed in 2 mL amber vials (2–3) capped with bonded pre-slit PTFE/silicone septum screw caps and placed in the cooled drawer.

**Laboratory supplies**

1. 2 mL amber vial (Agilent, catalog number: 5190-9063)

2. Bonded PTFE/silicone septum screw cap (Agilent, catalog number: 5190-9068)

3. Magnetic cap Silicone/PTFE/starburst-slitted septa (PAL, catalog number: Cap-ND9-St-SP10Sb-100)

4. Glass amber vial with 0.2 mL integrated insert (MACHEREY-NAGEL, catalog number: 702008)

5. 10 mL amber flat bottom glass vial (JG Finneran, catalog number: 31018F-2346A) with Screw cap, solid top with PTFE liner (Supelco, catalog number: 27163)

6. Bonded pre-slit PTFE/silicone septum screw cap (Agilent, 5185-5824)

7. 0.1–10 μL epT.I.P.S. Reloads (Eppendorf, catalog number: 022491504)

8. 2–200 μL epT.I.P.S. Reloads (Eppendorf, catalog number: 022491733)

9. 50 μL–1 mL epT.I.P.S. Reloads (Eppendorf, catalog number: 022491555)

10. 0.5–10 mL epT.I.P.S. Standard (Eppendorf, catalog number: 022492098)

11. Labels for vials

12. Whatman 903 cards (Merck, catalog number: WHA10531018)

## Equipment

1. 0.5–10 μL pipette (Eppendorf Research^®^ plus, catalog number: 3123000020)

2. 20–200 μL pipette (Eppendorf Research^®^ plus, catalog number: 3123000055)

3. 100–1,000 μL pipette (Eppendorf Research^®^ plus, catalog number: 3123000063)

4. 1,000–10,000 μL pipette (Eppendorf Research^®^ plus, catalog number: 3123000080)

5. 3 mm hole puncher

6. Centrifugal evaporator (SP Genevac EZ-2 Series centrifugal evaporator, model: 3.0, Elite)

7. Storage boxes for 2 mL vials (VWR, catalog number: 525-0934)

8. Mini vortex (Wizard D, catalog number: 444-0746)

9. AS X2 PLUS analytical balance (RADWAG, product code: WL-104-0191)

10. O-rings, Viton (Restek, catalog number: 22242)

11. Thermo Scientific^TM^ TriPlus^TM^ RSH with extended rail (Thermo, catalog number: 1R77010-0400)

12. Thermo Scientific Orbitrap^TM^ Exploris^TM^ GC 240 mass spectrometer (Thermo, catalog number: BRE725537)

13. TriPlus RSH, automatic tool change (ATC) station (Thermo, catalog number: 1R77010-1019)

14. 2 TriPlus RSH, liquid tool: D7, 57 mm (Thermo, catalog number: 1R77010-1007)

15. Triplus RSH autosampler syringe 57 mm, 23 s Ga, cone, 100 µL (Thermo, catalog number: 365H2141)

16. TriPlus RSH autosampler syringe 57 mm, 23s Ga, cone, 10 µL (Thermo, catalog number: 365D0311)

17. TriPlus RSH, agitator/incubator (Thermo, catalog number: 1R77010-1032)

18. TriPlus RSH, temperature-controlled drawer: single (Thermo, catalog number: 1R77010-1028)

19. Topaz Liner, splitless, gooseneck. w/wool 4 mm × 6.3 × 78.5 (Restek, catalog number: 23303)

20. Merlin microseal (Restek, catalog number: 22812)

21. Rxi-5Sil MS, 0.25 μm, 0.25 mm ID low polarity phase, 15 m length, 0.25 mm ID, 0.25 μm film thickness (Restek, catalog number: 13620)

22. Rxi guard column phase free, 0.53 mm ID, 2 m length to inlet (Restek, catalog number: 10073)

23. Rxi guard column phase free, 0.25 mm ID, 2 m length to spectrometer (Restek, catalog number: 10059)

24. Tweezers

## Software and datasets

1. Sampling Workflow Editor (SWE) (version 1.4.0.4)

2. Thermo Scientific Xcalibur (version 4.4.16.14, February 6, 2020)

3. Chromeleon 7.2.10

4. PALscript Editor (version 3.1 Beta)

5. EIRENE-CZ_RECETOX Metabolome high resolution–electron ionization–mass spectral library (HR-[EI+]-MS), free, CC-BY-NC, https://doi.org/10.5281/zenodo.5483565

6. MS-DIAL (version 4.9.221218), free, CC-BY 4.0, https://doi.org/10.5281/zenodo.12589462

7. Skyline (version 23.1.0.268), free, modified Apache 2.0 License, https://github.com/ProteoWizard/pwiz/tree/master/pwiz_tools/Skyline

8. Spreadsheet software, e.g., Microsoft Excel, Google Sheets (free under Google terms of service)

9. BioRender (https://www.biorender.com/). The following figures were created using BioRender: Graphical overview, https://BioRender.com/v67e825; Figure S1, https://BioRender.com/y04y703

## Procedure

**A. Sample preparation: manual steps**

**Sample preparation of dried blood spots**

1. Punch 3.3 mm from the DBS cards.

2. Place the punch using tweezers directly in a glass amber vial with 0.2 mL integrated insert and seal with magnetic cap silicone/PTFE/starburst-slitted septa.

**Sample preparation of liquid blood matrices**

3. Homogenize samples by gentle shaking and pipetting with ~5 inversions and 3 times pipetting (serum/plasma) or vortexing for ~10 s (HPLM).

4. Dispense 3 μL of liquid blood matrices in a glass amber vial with 0.2 mL integrated insert and seal with magnetic cap silicone/PTFE/starburst-slitted septa.

5. Dry for 20 min using the centrifugal evaporator, with the aqueous program with the lamp off.

**Sample preparation of blanks**

6. Prepare procedural blanks for DBS analysis by punching 3.3 mm from unused DBS cards into glass amber vials with 0.2 mL integrated insert and seal with magnetic cap silicone/PTFE/starburst-slitted septa. Use empty vials as procedural blanks for liquid blood samples and cap with magnetic cap silicone/PTFE/starburst-slitted septa.

7. Place vials containing samples, procedural blanks, and reagents (pyridine, Mono-Deca PCBs solution, C_7_-C_40_ alkane solution, MeOx solution, and MSTFA working solution) in the cooled drawer tray. Figure S1 shows an example of the tray layout.

*Note: Pyridine is used as instrumental blank; the Mono-Deca PCBs solution is used for system suitability testing, e.g., checking mass accuracy, retention, and ion abundance prior to analysis; and C_7_-C_40_ alkane solution is used to generate external non-isothermal Kováts retention index [19]. These solutions are directly injected, i.e., without automated sample preparation, as shown in Table S1.*

**B. Automated sample preparation**

The automated sample preparation is performed on a Thermo Scientific^TM^ TriPlus^TM^ RSH equipped with modules as displayed in Figure S2.

1. The automated method was created using SWE as detailed in standard operating procedure (SOP) (https://doi.org/10.5281/zenodo.10612856) and edited according to SOP (https://doi.org/10.5281/zenodo.10612909). The finalized method script is available in File S1, and a video simulating the method is available in File S2. Upload the method to the analytical sequence.

2. Run the sequence containing details about the injection list, instrumental method, sample position, and injection volume. Table S1 shows the sequence layout.

**C. GC-HRMS analysis**

Once sample preparation finishes, 1 μL of the derivatization product is automatically injected into the chromatographic system in splitless mode. The gas chromatography and mass spectrometry acquisition parameters are the same as previously provided [20,21].

In brief, analytes are separated upon a Restek Rxi5-Sil MS column (15 m, 0.25 mm ID, 0.25 μm) connected to a 2 m pre- and post-column. The GC oven temperature is initially set to 80 °C for 0.5 min, ramped to 200 °C at 40 °C/min with a hold time of 0.5 min¸ followed by a second ramp of 40 °C/min to 260 °C (also with 0.5 min hold), and then a third ramp of 55 °C/min to 330 °C with 4 min hold. The auxiliary temperatures are 280 °C. Helium is used as carrier gas at 1.2 mL/min. Electron ionization is performed at 70 eV with 50 μA emission current and 15 V electron lens. Data are acquired in profile mode, scanning 70–700 m/z at a resolving power of 60 K at 200 m/z. The SSL injector is equipped with a Merlin micro-seal and topaz liner. Exchange the topaz liner and O-ring for each sequence.

## Data analysis

**A. Metabolite composition analysis**

1. Non-targeted data processing is performed in MS-DIAL [22,23]. Settings for detection (peak picking), deconvolution, alignment, and identification for MS-DIAL are provided in Table S2. Import data in .raw profile format.

2. Manually check peak integrations in MS-DIAL and update where needed.

3. Export alignment results and select raw data matrix (area) and representative spectra.

4. Open the peak area report as a spreadsheet (.csv) and, per each feature, subtract the maximum value detected in any procedural blank from the respective samples measured in the same analytical batch. Subsequent analysis can be performed on the finalized peak area matrix and/or mass spectra (.msp).

**B. Selected metabolite quantification**

1. Peak picking and integration are performed using the molecule interface of Skyline [24,25]. Transition settings and molecule settings are provided in Table S3 and Table S4, respectively.

2. A transition list of 52 TMS derivatives, representing 34 selected metabolites plus the 3 deuterated internal standards, is provided in Table S5 for targeted integration. The transition list contains at least three ions selected per analyte. Ions and retention time windows were selected based upon analysis of reference standards per each analyte [included in the EIRENE-CZ_RECETOX metabolome high resolution–electron ionization–mass spectral library (HR-[EI+]-MS)]. When possible, the theoretical masses of ions are used based on fragment formula annotation [26].

3. Import data in .raw format, manually check, and update peak integrations.

4. Export report for molecular transition results containing integrated peak areas per ion.

5. Open the molecular transition report as a spreadsheet (.csv) and select a quantitative ion per metabolite, e.g., select the ion showing the lowest coefficient of variation (CV).

6. Sum the integrated peak areas of the chosen quantification ions for all derivatives of an analyte [14].

7. Calculate the CVs per analyte quantitation based on the summed data. Subsequent analysis can be performed on the finalized metabolite area dataset.

## Validation of protocol

The method was applied to the various blood samples, i.e., DBS capillary, DBS venous, serum, and plasma, with each matrix being analyzed in six replicates. For DBS samples, the six punches came from six different spots collected from an individual’s single timepoint blood draw. Raw files and Skyline processing are available on Panorama Public [27,28] at https://doi.org/10.6069/aeg8-9065.

The average CV across blood sample types was 2% for D_27_-myristic acid (1TMS derivative), 2% for D_5_-glycine (3 TMS derivative), and 4% for D_7_-cholesterol (1TMS derivative), evidencing high reproducibility of the automated sample preparation.

**Figure 1.**
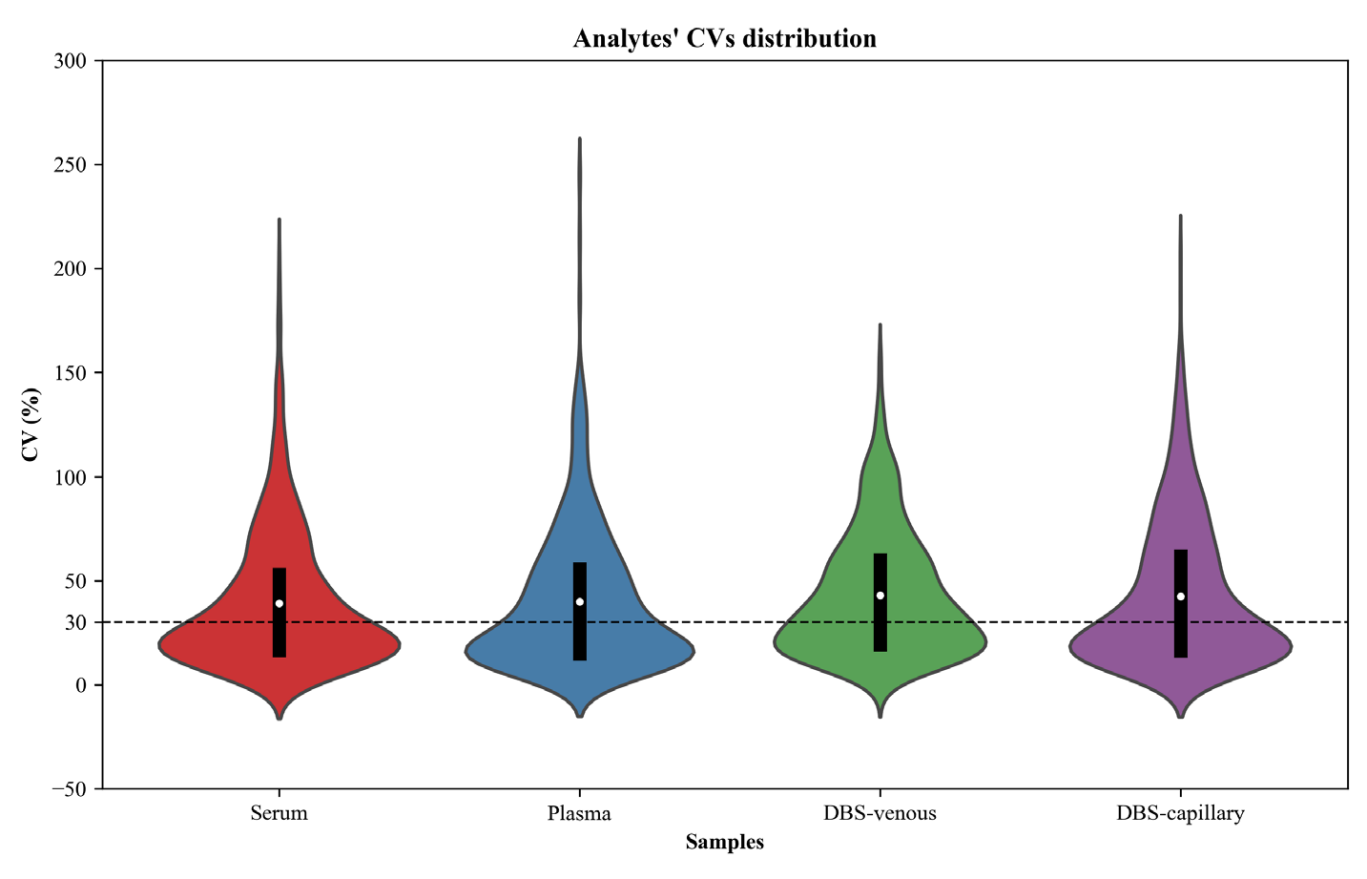
Violin plots of the coefficients of variation (CV) for peak area of features detected following GC-Orbitrap MS metabolomics analysis of various blood samples. Serum, plasma, DBS capillary, and DBS venous samples were analyzed in six replicates. A greater proportion of features are reproducibly measured, i.e., below the 30% CV threshold (dashed line), in liquid blood samples. The median is indicated by a white dot and the interquartile range (IQR) by a black box.

Analysis of generated data via MS-DIAL led to the detection of >600 deconvoluted features per matrix. Using a threshold of 30% CV [3], 449, 450, 264, and 329 features are reproducibly measured in serum, plasma, DBS venous, and DBS capillary, respectively (Figure 1). Over 70 analytes had confirmed identification for each matrix, i.e., through comparison with the RECETOX Metabolome HR-[EI+]-MS library (Table 1, Table S6). Analytes reported with higher confidence in annotation showed lower relative standard deviation across replicates, likely owing to a positive correlation between analyte abundance, peak, and spectral quality.

**Table 1.** Summary of deconvoluted features per blood matrix

Sample	Features number	All features mean average CV (%)	Identified* analytes	Identified analytes mean average CV (%)
Serum	839	39	77	22
Plasma	889	40	75	19
DBS venous	626	43	70	25
DBS capillary	650	43	69	23

*Analytes are annotated by comparison with RECETOX Metabolome HR-[EI+] MS library.

**Figure 2.**
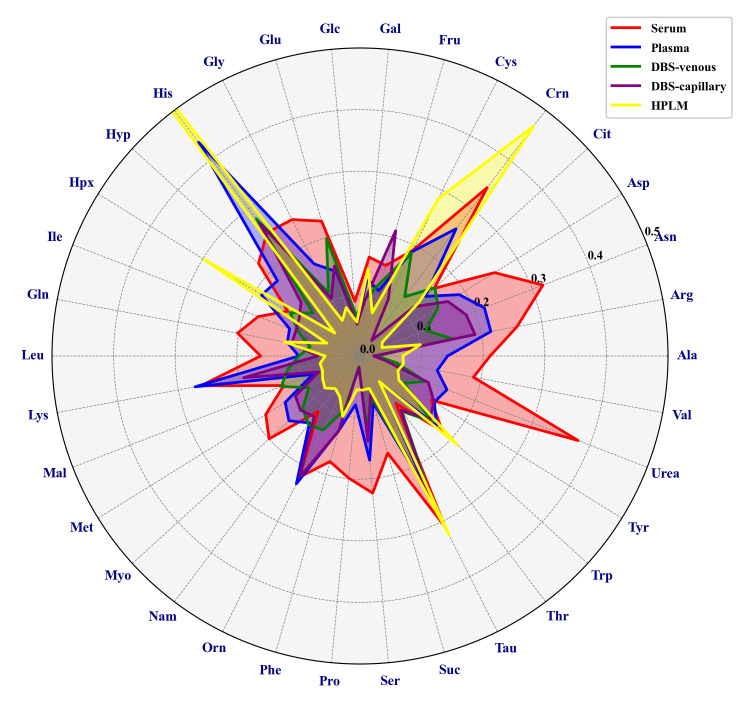
Radar chart showing the coefficients of variation (CVs) of selected metabolites quantified by GC-Orbitrap MS metabolomics analysis of various blood samples. Serum, plasma, DBS capillary, DBS venous, and HPLM samples were analyzed in six replicates. When analytes have multiple derivatives, peak areas have been summed. Alanine (Ala), Arginine (Arg), Asparagine (Asn), Aspartic acid (Asp), Ornithine (Orn), Citric acid (Cit), Cysteine (Cys), Glutamic acid (Glu), Glycine (Gly), Histidine (His), Hydroxyproline (Hyp), Isoleucine (Ile), Glutamine (Gln), Leucine (Leu), Lysine (Lys), Threonine (Thr), Methionine (Met), Phenylalanine (Phe), Proline (Pro), Serine (Ser), Taurine (Tau), Tryptophan (Trp), Tyrosine (Tyr), Valine (Val), Creatinine (Crn), Fructose (Fru), Glucose (Glc), Galactose (Gal), Hypoxanthine (Hpx), Malic acid (Mal), Myoinositol (Myo), Niacinamide (Nam), Succinic acid (Suc), Urea (Urea).

A selective target integration of a subset of 34 confirmed analytes also present in HPLM [as a surrogate certified reference material (CRM) material] was performed via Skyline. Multiple features derived from a single analyte are summed in order to check quantitative reproducibility [14]. The CVs for metabolites are below 30% except for asparagine, creatinine, taurine, and urea in serum; histidine in plasma; and creatinine, histidine, hypoxanthine, and taurine in HPLM (Figure 2).

## General notes and troubleshooting

**General notes**

1. The workflow of sampling preparation was created and edited using Sampling Workflow Editor; however, the workflow was further optimized and improved using PALscript Editor.

2. Thermo Scientific^TM^ GC-MS system equipped with Thermo Scientific^TM^ TriPlus^TM^ RSH needs to be configured for manipulation under Xcalibur or Chromeleon before running sample preparation and analysis (for that, use Thermo Foundation Instrument to configure the different compartments of the instrument under Xcalibur or Chromeleon Services Manager to configure under Chromeleon).

3. The derivatization method can also be applied to dried extracts and other materials, e.g., dried urine and seminal plasma samples (as per https://doi.org/10.5281/zenodo.7462217 [21] and https://doi.org/10.5281/zenodo.5734331 [20], respectively).

4. Alternatively, internal standards can be added directly to the vial containing MeOX solution. We used D_5_-glycine, D_27_-myristic acid, and D_7_-cholesterol because they elute at the beginning, middle, and end of the chromatogram and represent different compound classes.

5. The blood collected here was from adults, but collection can be applied to children and neonates. Different DBS sampling cards can be used.

6. HPLM was used as a surrogate certified reference material, but alternative materials can be used, e.g., NIST Standard Reference Material (SRM) 1950.

7. Tutorial for MS-DIAL data processing: https://systemsomicslab.github.io/mtbinfo.github.io/MS-DIAL/tutorial

8. Tutorial for Skyline (Hi-Res Metabolomics) data processing: https://skyline.ms/wiki/home/software/Skyline/page.view?name=tutorial_hi_res_metabolomics

9. Other libraries can be used for spectral matching, e.g., NIST/EPA/NIH Mass Spectral Library (proprietary).

10. The Thermo Scientific^TM^ TriPlus^TM^ RSH protocol can be coupled to the analysis of sample extracts via any coupled GC–MS model (e.g., single quadrupole, time of flight), yet analyte coverage and detection will depend on GC–MS methodology.

11. The procedure can be adapted to comparable cartesian autosamplers (e.g., PAL RTC, Gerstel MPS) if parameters can be mapped to the respective instrument control software.

**Troubleshooting**

1. We recommend the use of PAL magnetic caps to avoid collisions.

2. If 2 mL vials are transported, it is recommended to remove the magnetic transport ring (adapter) that is used in the case of 20/10 mL vials.

3. Cleaning the syringe needle and plunger used for sample preparation (here, 100 μL syringe) is recommended after running the sequence to avoid crystallization of the reagent. Wipe the plunger clean with a lint-free tissue, taking care not to bend the plunger. Remove the plunger and fill the syringe with solvent, insert the plunger back, and gently push the solvent through the needle. In this application, methanol and acetone were used as solvents for cleaning.

4. Pre-slitted caps are recommended for samples and reagents that will be penetrated multiple times.

## Supplementary information

The following supporting information can be downloaded here:

1. Figure S1. Layout of reagents and samples in VT 54 cooled drawer trays. Example of sequence is provided in Table S1 for 24 h operation. Created in BioRender. Omics, M. (2024) https://BioRender.com/y04y703.

2. Figure S2. Thermo Scientific^TM^ TriPlus^TM^ RSH autosampler configuration. 1). X-axis extended rail with built in electronic and control. 2). Autosampler’s head with Z-axis. 3). Automatic tool change (ATC) station equipped with liquid handling tools. 4). Large wash station: 2 × 200 mL vials and a waste port. 5). Cooler drawer for controlled cool temperature and sealed under nitrogen: 2 × VT54 trays. 6). Standard wash station: 5 × 10 mL vials. 7). Agitator with controlled temperature for incubation with gentle shaking 6 positions adaptors installed for 2 mL vials.

3. Table S1. Example sequence layout for 24 h operation.

4. Table S2. MS-DIAL parameters.

5. Table S3. Skyline transition settings.

6. Table S4. Skyline molecule settings.

7. Table S5. Skyline transitions list to quantify select metabolites.

8. Table S6. List of analytes identified in blood samples.

9. File S1. Autosampler method script (.xml).

10. File S2. Video showing simulated sample preparation.
